# Gold Nanoparticle Complexes with PAMAM Dendrimers for In Vitro Cancer Cytotoxicity Assessment: Synthesis via Ascorbic Acid Reduction

**DOI:** 10.3390/molecules31111844

**Published:** 2026-05-27

**Authors:** Agnieszka Maria Kołodziejczyk, Bolesław T. Karwowski, Magdalena Grala

**Affiliations:** 1 Food Science Department, Faculty of Pharmacy, Medical University of Lodz, Muszynskiego 1, 90-151 Lodz, Poland; agnieszka.kolodziejczyk@umed.lodz.pl; 2 Department of Chemical and Molecular Engineering, Faculty of Process and Environmental Engineering, Lodz University of Technology, Wolczanska 213, 93-005 Lodz, Poland; magdalena.grala@p.lodz.pl

**Keywords:** synthesis of gold nanoparticles, PAMAM G2, AuNPs/PAMAM G2 complexes, XTT cytotoxicity tests

## Abstract

Ascorbic acid plays an important role in the human body due to its antioxidant and anti-inflammatory properties, as well as its involvement in collagen synthesis, enzymatic regulation, and the biosynthesis of corticosteroids and selected neurotransmitters. Owing to these diverse functions, it is used both in the prevention and supportive treatment of several disorders and as a mild, non-toxic reducing agent in the synthesis of gold nanoparticles (AuNPs). In the present study, a method for synthesizing gold nanoparticles was developed using second-generation poly(amidoamine) dendrimers (PAMAM G2) with an ethylenediamine core as stabilizing agents and ascorbic acid as the reducing agent. The synthesis was performed using two techniques: sonication and microwave irradiation. A comparative analysis was conducted for colloidal systems obtained at various molar ratios of PAMAM G2 dendrimers to chloroauric acid (ranging from 1:1 to 1:5). The presence of gold nanoparticles was confirmed using ultraviolet–visible spectroscopy (UV–Vis). Nanoparticle diameters and zeta potentials were determined by dynamic light scattering (DLS). The sizes of the metallic cores were estimated using scanning transmission electron microscopy (STEM). Furthermore, the morphology and topography of entire complexes deposited on silicon substrates were visualized using atomic force microscopy (AFM). For cytotoxicity studies on human breast adenocarcinoma and human osteosarcoma cell lines, the most stable colloids—those obtained at a PAMAM G2:HAuCl_4_ molar ratio of 1:3—were selected. Results indicate that the synthesized nanoparticles exhibit slightly higher cytotoxicity compared with AuNPs/PAMAM G2 complexes reduced with sodium citrate, as evidenced by lower EC_50_ values (the concentration responsible for reducing cell viability to 50%). It should be emphasized, however, that AuNPs/PAMAM G2 reduced with ascorbic acid are significantly smaller, with diameters of approximately 10 nm, whereas citrate-reduced nanoparticles exhibit diameters of around 20 nm. These results indicate that nanoparticle size, rather than the chemical nature of the reducing agent, is a dominant factor governing the cytotoxic response of AuNPs/PAMAM G2 complexes.

## 1. Introduction

Over the past few years, the synthesis of nanoparticles has increasingly shifted away from conventional methods that rely on toxic and hazardous reagents toward the development of more biocompatible and environmentally friendly alternatives [1,2]. This aspect is particularly important for the synthesis of nanoparticles intended for applications in biological and medical sciences [3]. In this context, the use of *L*-ascorbic acid (vitamin C) as a reducing agent appears to be particularly well suited to green synthesis strategies, providing a more environmentally friendly and less toxic alternative to conventional reducing agents.

*L*-ascorbic acid (vitamin C) is an unsaturated polyhydroxy alcohol and exhibits good solubility in water as well as in ethanol, glycerol, and propylene glycol [4]. Despite its important biological role, the human body does not synthesize vitamin C endogenously; therefore, it must be supplied through the diet [5]. One of the key functions of *L*-ascorbic acid is its antioxidant activity—the neutralization of reactive oxygen species (ROS) [6,7]—thereby reducing oxidative stress and protecting cells [8].

In recent years, numerous studies have described the use of ascorbic acid as both a reducing and stabilizing agent in the synthesis of metallic nanoparticles, in particular gold nanoparticles (AuNPs) [9,10]. The mechanism of complexation between gold ions and ascorbate, as well as the formation of AuNPs, was thoroughly examined by Zumreoglu Karan [11]. Malassis et al. [9] proposed a one-step, rapid, and environmentally friendly method for synthesizing AuNPs using ascorbic acid (AA). Although the resulting AA AuNP colloids exhibited polydispersity (10–50%), they retained broad application potential due to the possibility of post-synthetic surface modification with surfactants of various charges. Jayeoye et al. [10] further demonstrated that vitamin C can simultaneously act as both a reducing and stabilizing agent and can facilitate the reduction of Ag^+^ ions on the nanoparticle surface, as confirmed by the shift in the SPR maximum from 522 to 400 nm. Hussain et al. [12] investigated the synthesis of gold nanoparticles in reaction media with varying polarity, using *L*-ascorbic acid as the reducing agent and polyvinylpyrrolidone as the stabilizer. Their analysis revealed that a decrease in solvent polarity (from ~8.2 to ~5.2) was accompanied by an increase in the hydrodynamic diameter of AuNPs (from ~22 to 219 nm). Higher polarity favored the formation of spherical nanoparticles, whereas lower polarity promoted the generation of larger particles with diverse morphologies [12].

The use of polyamidoamine (PAMAM) dendrimers as stabilizing agents and *L*-ascorbic acid as a reducing agent represents an attractive approach for the synthesis of gold nanoparticles. PAMAM dendrimers have been extensively investigated for their potential medical applications [13]. Numerous PAMAM dendrimer-based systems are currently in the clinical research stage or are incorporated into commercially available products as carriers for drugs, vaccines, and genetic material. Examples include gene delivery vectors such as SuperFect^®^ (Qiagen, Venlo, The Netherlands) and PrioFect^®^ (Qiagen, Venlo, The Netherlands) [14], the DNA transfection reagent PolyFect^®^ (Qiagen, Venlo, The Netherlands) [15], as well as OP-101 (Orpheris, Redwood City, CA, USA) [16], a formulation currently undergoing Phase I clinical trials. In addition, the compound [^18^F]OP-801 is being explored for applications in positron emission tomography (PET) [17]. PAMAM dendrimers also represent a promising platform as boron carriers, particularly after functionalization with boron cage structures, which creates opportunities for their potential use in boron neutron capture therapy (BNCT) [18]. However, it should be emphasized that the application of PAMAM dendrimers is limited by their inherent toxicity [19], which depends on the type of surface functional groups and the dendrimer generation [20,21]. Consequently, numerous strategies have been developed to reduce their cytotoxicity, including surface modifications through functionalization with various polymers, ligands, or antibodies [22,23].

PAMAM dendrimers are monodisperse, highly branched macromolecules with a spherical architecture [24], capable of encapsulating and stabilizing metallic nanoparticles [25]. Complexes of AuNPs/PAMAM exhibit significant potential as contrast agents for computed tomography (CT). Xiao et al. [26] demonstrated that Au/PAMAM G5 nanoparticles exhibit greater X-ray attenuation compared to the clinically used iodinated contrast agent Omnipaque at the same molar concentration of active elements (Au or I), enabling clearer in vivo imaging of rat hearts. Liu et al. [27] subsequently showed that AuNPs/PAMAM G2 provide radiation attenuation comparable to Omnipaque and improved visualization of major rat organs in vivo, likely due to their nanometric size (~6 nm) and prolonged circulation time. Sutriyo et al. [28] reported the synthesis of Au/PAMAM G4 using sodium borohydride as a reducing agent, where the resulting nanoparticles exhibited higher Hounsfield Unit (HU) values than iodinated contrast agents at equivalent concentrations.

**Figure 1:**
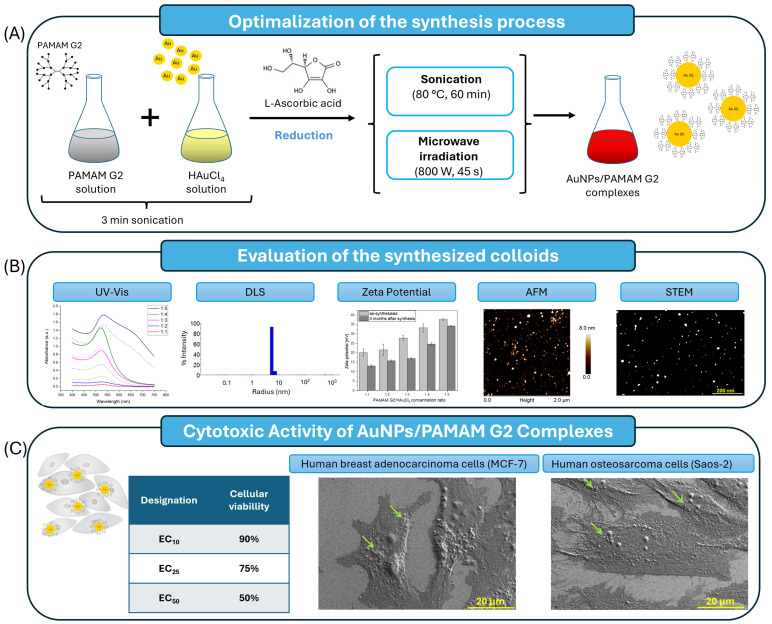
The schematically illustrated idea of the study is presented, followed by synthesis methods, including sonication and microwave irradiation and AuNPs/PAMAM G2 complex formation (**A**), evaluation of the physicochemical parameters of obtained complexes, (**B**) and cytotoxic effect of these complexes on human breast adenocarcinoma (MCF-7) and human osteosarcoma (Saos-2) cells along with the investigation of the presence and localization of the complexes (green arrows) on the cell membrane obtained by SEM imaging (**C**). The synthesis method was optimized for molar ratios of PAMAM G2:HAuCl_4_ ranging from 1:1 to 1:5 and was performed using sonication and microwave irradiation. The examples of AFM topography and STEM images of a 1:3 molar concentration ratio of PAMAM G2:HAuCl_4_ are presented (**B**). Additionally, the representatives of UV–Vis, DLS, and zeta potential data are shown (**B**). EC_10_, EC_25,_ and EC_50_ correspond to the 90, 75, and 50% of cellular viability in comparison to the negative control, respectively.

Gold nanoparticles possess advantageous biocompatibility arising from their chemical and physical stability, as well as their ability to be functionalized with a wide range of biologically active molecules [29]. They can form conjugates and interact with proteins [30], drugs [31], antibodies [32], enzymes, nucleic acids [33], and fluorescent dyes [34], which makes them attractive candidates for biomedical and biotechnological applications [35]. The aim of this study was to develop a rapid and reproducible method for synthesizing gold nanoparticles stabilized with second-generation PAMAM dendrimers (PAMAM G2), using *L*-ascorbic acid as the reducing agent, which constitutes the novelty of the manuscript. The presented studies are a continuation of our previous work [25,36,37]. The use of L-ascorbic acid as a reducing agent in the synthesis of AuNPs/PAMAM G2 complexes improved the colloidal stability over a period of 3 months compared to previous studies employing sodium citrate. The characteristics of the obtained AuNPs/PAMAM G2 complexes were determined using a range of microscopic and spectroscopic methods. In addition, the colloids were examined using scanning transmission electron microscopy (STEM), which allowed for a detailed analysis of the metallic core diameter. The scheme of the goal of the study is presented in Figure 1.

In previous work, sodium citrate was used as the reducer [36,37]; however, due to potential biomedical applications—and thus the need to minimize cytotoxicity—vitamin C was introduced as an alternative reducing agent (Figure 1A). Characterization of the obtained nanoparticles (Figure 1B) included evaluation of monodispersity and long-term stability over several months using techniques such as dynamic light scattering (DLS), zeta potential measurements, ultraviolet–visible spectroscopy (UV–Vis), and atomic force microscopy (AFM). The cytotoxicity of the AuNPs/PAMAM G2 complexes was assessed in MCF-7 (breast cancer) and Saos-2 (osteosarcoma) cell lines using the 2,3-bis[2-methoxy-4-nitro-5-sulfophenyl]-2H-tetrazolium-5-carboxanilide (XTT tetrazolium) reduction assay (Figure 1C). The presence and localization of AuNPs/PAMAM G2 complexes on the cell membrane were investigated using scanning electron microscopy (SEM).

## 2. Results and Discussion

### 2.1. Synthesis of AuNPs/PAMAM G2 with L-Ascorbic Acid as a Reducing Agent

The synthesis strategy of AuNPs/PAMAM G2 is based upon our previously reported model [36], with the key modification in the present study, i.e., the use of *L*-ascorbic acid as a reducing agent. In an aqueous medium, gold nanoparticles are formed by combining HAuCl_4_ with a second-generation poly(amidoamine) dendrimer (PAMAM G2) containing an ethylenediamine core. The ability of PAMAM dendrimers to stabilize AuNPs has been widely documented in the literature [38,39]. Molar ratios of PAMAM G2 to HAuCl_4_ were adjusted between 1:1 and 1:5 to investigate their influence on nanoparticle formation and stability. Due to electrostatic interactions, negatively charged AuCl_4_^−^ ions are attracted to protonated amine groups (–NH_3_^+^), located both on the dendrimer surface and within its interior, which may facilitate their spatial confinement within the dendrimer structure. Subsequent addition of *L*-ascorbic acid triggers the reduction of Au(III) to Au(0), initiating nucleation followed by nanoparticle growth. Elevated temperature (80 °C) and ultrasonic irradiation (sonication) or microwave irradiation enhance nucleation kinetics and promote more uniform particle formation. High temperature and ultrasonic irradiation are commonly used in classical synthesis methods for the preparation of metallic nanoparticles. These approaches are well established in the literature and allow the production of nanoparticles with controlled size and morphology [40]. Ultrasonic irradiation promotes mixing and enhances mass transfer in the reaction medium, which may facilitate nucleation and improve the uniformity of the obtained nanoparticles [41]. However, conventional heating methods typically require longer reaction times and may lead to temperature gradients within the reaction mixture. To overcome these limitations, alternative energy sources such as microwave irradiation have been increasingly applied in nanoparticle synthesis. Microwave irradiation enables rapid penetration of electromagnetic energy into the entire volume of materials that are transparent to microwave radiation, leading to fast and homogeneous heating of the reaction mixture [42]. As a result, microwave-assisted synthesis often provides shorter reaction times, improved control over nucleation and growth processes, and reduced energy consumption compared with conventional heating methods [43]. Thus, we decided to compare the possibilities of obtaining stable nanoparticles in a controlled and reproducible manner using two methods: the traditional sonication technique and the faster microwave method. Consequently, the final characteristics of the synthesized nanoparticles depend not only on the applied energy source but also on the chemical environment of the reaction medium. In particular, macromolecular stabilizers such as dendrimers may significantly influence both the nucleation stage and the stabilization of the formed nanoparticles. The preliminary concentration of gold ions by the dendrimer promotes the formation of uniform nanoparticles. Li et al. [39] demonstrated that once the metallic core is formed, the surface of the AuNPs becomes coated with PAMAM molecules, a phenomenon characteristic of lower-generation dendrimers. According to our previous findings, the primary mechanism of direct chemical attachment involves the formation of stable coordination bonds between gold atoms on the nanoparticle surface and nitrogen atoms from the dendrimer’s amine groups (–NH_2_, –NH, or –N). These bonds play a crucial role in surface stabilization and prevention of immediate aggregation. Camarada et al. [38] showed that long-term stability is additionally governed by non-covalent interactions, with steric stabilization provided by the branched dendrimer architecture and electrostatic stabilization originating from the terminal amine groups.

### 2.2. Physicochemical Parameters of Gold Nanoparticle Colloids Stabilized by PAMAM Dendrimers

**Figure 2:**
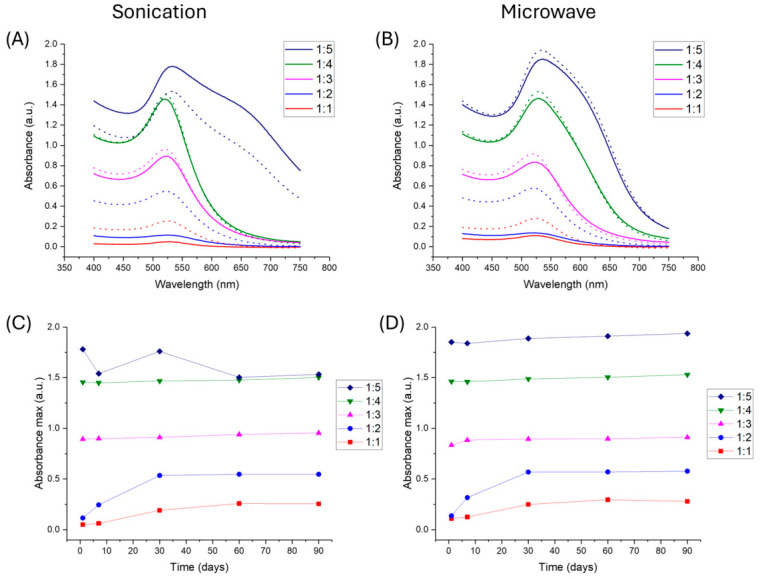
UV–Vis spectra of Au/PAMAM complexes of 1:1, 1:2, 1:3, 1:4, and 1:5 samples synthesized using sonication (**A**), microwaves (**B**), and absorbance maximum values for all samples after 24 h, 7 days, 1 month, 2 months, and 3 months for both sonication (**C**) and microwave (**D**) methods. The selected colors indicated the samples as follows: red (1:1), blue (1:2), pink (1:3), green (1:4), dark blue (1:5). Solid curves represent the spectra collected 24 h after synthesis, whereas dashed lines indicate measurements made after 3 months.

The AuNPs/PAMAM G2 complexes, designated as 1:1, 1:2, 1:3, 1:4, and 1:5, were confirmed using UV–Vis spectroscopy. The analysis was performed after 24 h, 1 week, 1 month, 2 months, and 3 months. Figure 2A,B present the UV–Vis spectra obtained for all samples at the 24 h and 3-month time points, whereas Figure 2C,D summarize the absorbance maxima for all samples across all UV–Vis measurement intervals.

The presence of AuNPs/PAMAM G2 complexes was confirmed by the appearance of an absorption band in the 525–550 nm range. As expected, the absorbance increases with increasing HAuCl_4_ concentration. In the case of the 1:5 sample, a broader absorption band in the 620–720 nm range was observed, indicating the formation of agglomerates of these complexes. For the sonication method, the 1:3 and 1:4 samples (pink and green lines in Figure 2C) exhibit the highest colloidal stability over time, maintaining high absorbance intensities. In the case of microwave synthesis, the 1:3, 1:4, and 1:5 samples (pink, green, and dark blue lines in Figure 2D) show the greatest stability over time. The wavelengths at which maximum absorbance was observed after 24 h were 522 nm (1:3, sonication), 521 nm (1:4, sonication), 522 nm (1:3, microwave), 528 nm (1:4, microwave), and 535 nm (1:5, microwave).

Hydrodynamic diameter values of the AuNPs/PAMAM G2 colloids, together with the corresponding mass fractions of individual populations, obtained for samples synthesized using sonication and microwave methods, are presented in Table 1 and Table 2, respectively.

DLS measurements were performed for samples with molar ratios ranging from 1:1 to 1:5 at time intervals of 24 h, 1 week, 1 month, 2 months, and 3 months after synthesis. For the 1:1 sample obtained via sonication, a small fraction of AuNPs/PAMAM G2 with a diameter of approximately 17 nm was observed immediately after synthesis, as confirmed by both the low mass fraction (2.3%) and the low absorbance value (Figure 2, red line). After one month, the sample exhibited a monodisperse distribution and significantly higher maximum absorbance values, whereas after three months, nanoparticle agglomeration was observed. For the 1:2 and 1:3 samples (sonication), similar hydrodynamic diameters of approximately 11 nm were obtained, with stable maximum absorbance values maintained only for the 1:3 sample (Figure 2C). Additionally, the 1:2 and 1:3 colloid obtained using the microwave method was stable and monodisperse (up to 2 months), with an average nanoparticle diameter of approximately 11 nm and 10 nm, respectively. However, the 1:3 samples prepared by both sonication and microwave synthesis remained stable, with nanoparticle agglomeration observed only after 2 months. For both synthesis methods, the 1:4 and 1:5 samples exhibited polydisperse characteristics, i.e., the AuNPs/PAMAM G2 colloids contained two or more distinct nanoparticle populations.

In summary, all colloids obtained at molar ratios ranging from 1:1 to 1:5 were suitable for characterization using the DLS technique. Monodisperse nanoparticles, which maintained a stable size distribution for up to three months after synthesis, were produced at a PAMAM G2-to-HAuCl_4_ molar ratio of 1:3; in this case, the hydrodynamic diameter was approximately 11 nm. Under sodium citrate reduction conditions [36], and in the sonication-assisted synthesis procedure, monodispersity was achieved only at a molar ratio of 1:4, with the resulting nanoparticles exhibiting a diameter of approximately 20 nm. Notably, when sodium citrate reduction was combined with the sonication-assisted method, no monodisperse size distribution was observed in any of the analyzed samples [36].

**Figure 3:**
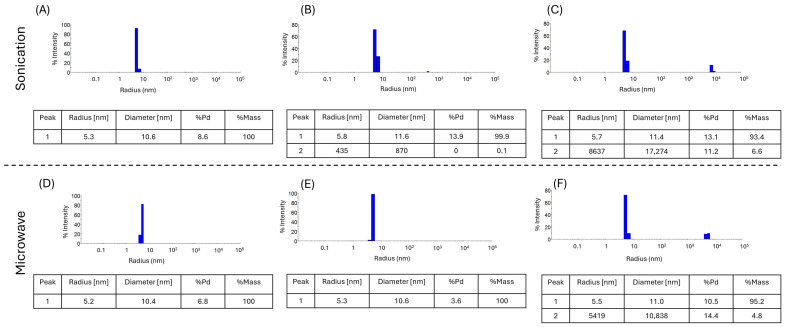
Examples of DLS size distributions of AuNPs/PAMAM at a 1:3 PAMAM G2:HAuCl_4_ concentration ratio measured 24 h (**A**,**D**), 2 months (**B**,**E**), and 3 months (**C**,**F**) after synthesis. The upper histograms correspond to the sonication method, whereas the lower histograms correspond to the microwave method. Tables placed below the histograms provide the identification of nanoparticle populations within the colloid, including their hydrodynamic diameters, corresponding polydispersity percentages (%Pd), and mass percentages (%Mass).

Figure 3 presents representative apparent hydrodynamic size distributions of AuNP colloids at a 1:3 PAMAM G2:HAuCl_4_ concentration ratio for both synthesis methods. Notably, for both methods, monodisperse gold nanoparticles with hydrodynamic diameters of approximately 10 nm were obtained, as confirmed by DLS measurements after 24 h. Furthermore, after 2 months, the microwave-synthesized nanoparticles remained monodisperse (Figure 3E), whereas in the sonication-assisted synthesis, slight agglomeration of AuNPs/PAMAM G2 was detectable (0.1% mass; Figure 3B). The main gold nanoparticle population remained unchanged after 3 months for both synthesis methods, although agglomeration and aggregation of AuNPs with sizes exceeding the micrometer range were observed (Figure 3C,F).

**Figure 4:**
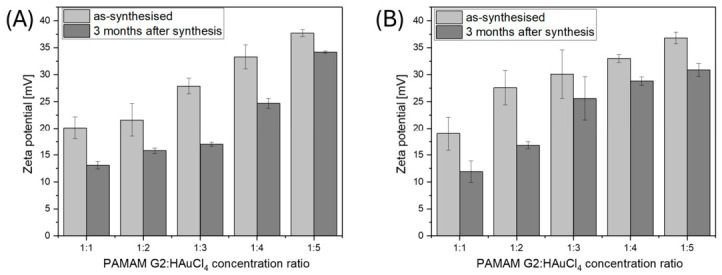
Zeta potential values for AuNPs/PAMAM G2 samples synthesized via sonication (**A**) and microwave (**B**) synthesis.

Figure 4 presents zeta potential values for AuNPs/PAMAM G2 samples with PAMAM-to-HAuCl_4_ molar ratios ranging from 1:1 to 1:5, synthesized using sonication (A) and microwave (B) methods, measured immediately after synthesis and after a three-month storage period. The recorded values, ranging from 10 to 40 mV, exhibit a clear dependence on the concentration of the HAuCl_4_ precursor. For both synthesis methods, a systematic decrease in zeta potential was observed across all samples, with percentage decreases (ranging from 10% to 39%—Table 3) observed after three months of storage. This reduction suggests that time-dependent modifications occur at the surface of the AuNPs/PAMAM G2 complexes during storage. The most plausible mechanism is the neutralization of residual positively charged dendrimer groups by remaining *L*-ascorbic acid ions present in the colloidal system.

**Figure 5:**
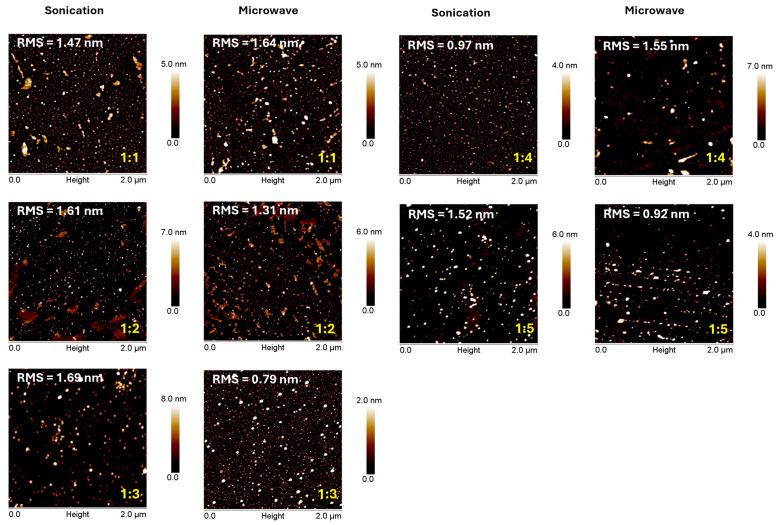
Examples of surface topography images of AuNPs/PAMAM G2 synthesized via sonication and microwave-assisted methods and deposited onto silicon substrates. Scan area: 2 × 2 μm; resolution: 512 × 512 pixels.

Figure 5 shows the AFM topography images of AuNPs/PAMAM samples deposited on silicon substrates (scan area: 2 × 2 μm). Samples with molar ratios of 1:1 and 1:2 exhibited predominantly individual nanoparticles together with some larger structures, attributed to agglomerates or aggregates. The 1:3 sample and 1:4 sample synthesized via sonication were characterized by the highest homogeneity. This homogeneity refers to the uniformity of the individual samples mentioned. In contrast, the 1:4 (microwave-assisted) and 1:5 samples revealed mainly larger surface structures. The root mean square (RMS) roughness values were below 2 nm for all samples, indicating low surface roughness and suggesting that the observed agglomerates or aggregates were small and not dominant.

Due to their stability over a three-month period, evidenced by the unchanged maximum absorbance values (Figure 2C,D), the presence of only minor agglomerate fractions after three months (Figure 3C,F), and the comparable hydrodynamic diameters of approximately 11 nm—the 1:3 colloids—were selected for STEM analysis and for cytotoxicity studies in the MCF-7 and Saos-2 cell lines.

**Figure 6:**
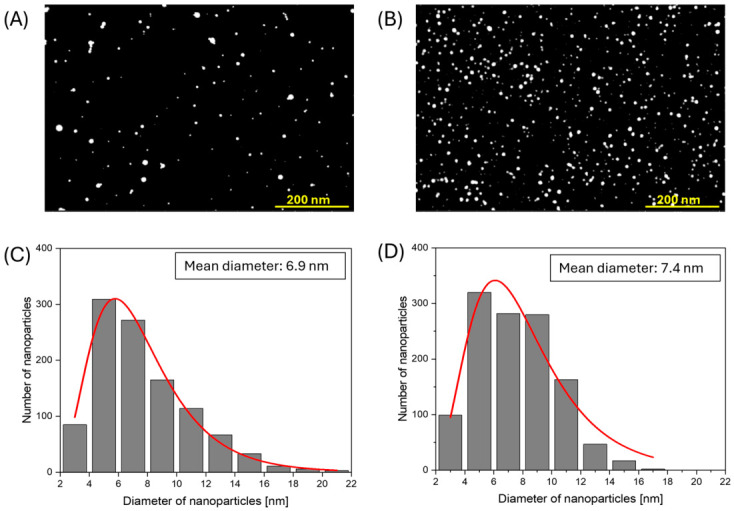
STEM images of AuNPs/PAMAM samples with a molar ratio of 1:3 synthesized via sonication (**A**) and microwave irradiation (**B**), along with particle size distribution histograms for nanoparticles synthesized using sonication (**C**) and microwave methods (**D**).

The STEM images (Figure 6A,B) show spherical, well-dispersed nanoparticles in the 1:3 samples, synthesized by both sonication and microwave techniques. Statistical analysis, presented in the histograms (Figure 6C,D), was performed on over 1000 nanoparticles per sample. The mean particle diameter was 6.9 nm and 7.4 for nanoparticles synthesized via sonication and microwave-assisted synthesis, respectively. Slightly smaller particle sizes obtained from STEM compared to DLS are expected, as DLS measures the hydrodynamic diameter, which includes the inorganic core along with surface ligands and the solvation shell, whereas STEM measures only the dehydrated nanoparticle core.

### 2.3. Cytotoxic Activity of AuNPs/PAMAM Complexes

**Figure 7:**
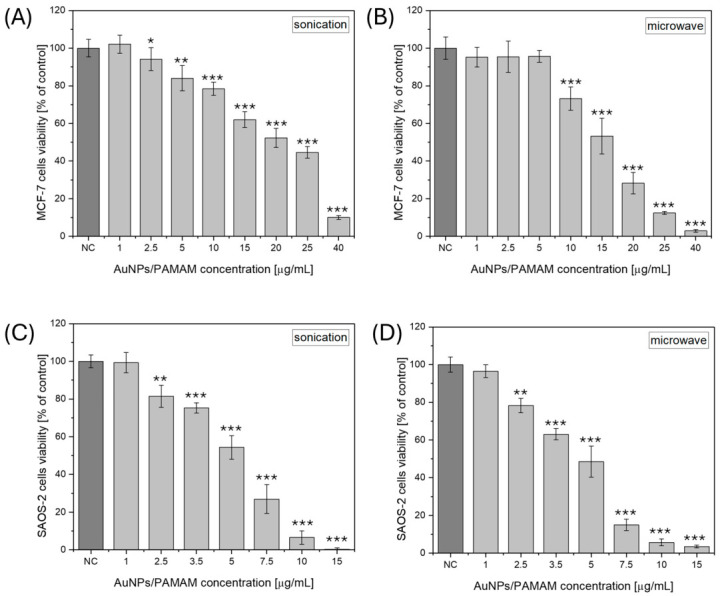
Viability of MCF-7 (**A**,**B**) and Saos-2 (**C**,**D**) cells investigated by the XTT test after incubation with AuNPs/PAMAM G2. NC refers to negative control (i.e., untreated cells). Statistical significance versus respective negative control is indicated when appropriate (* *p* < 0.05, ** *p* < 0.001, *** *p* < 0.0001).

Figure 7 illustrates the metabolic activity of Saos-2 and MCF-7 cells following treatment with selected 1:3 AuNPs/PAMAM G2 formulations. The dataset integrates results obtained for nanoparticles synthesized using two distinct approaches—sonication and microwave irradiation—allowing for a direct comparison of their biological effects. A clear concentration-dependent decline in cell viability was observed for both cell lines. Notably, a statistically significant cytotoxic response was observed at concentrations exceeding 2.5 µg/mL for both cell lines, as confirmed by ANOVA analysis (* *p* < 0.01, ** *p* < 0.001, *** *p* < 0.0001).

When comparing the present results with our previous findings [36], AuNPs/PAMAM G2 toxicities (Table 4) are at similar levels (EC_10_, EC_25_, EC_50_). It should be noted, however, that in the earlier study [36], where gold reduction was carried out in the presence of sodium citrate, the nanoparticles exhibited a two-fold higher hydrodynamic radius (sonication method) and two population sizes of approximately 9 and 100 nm (microwave method).

### 2.4. Size-Dependent Cytotoxicity of Ascorbic-Acid-Reduced AuNPs

Cytotoxic effects observed for AuNPs/PAMAM G2 reduced with *L*-ascorbic acid correspond well to the size-dependent toxicity trends widely reported in the literature for gold nanoparticles. Numerous studies have demonstrated that AuNPs with diameters below approximately 10 nm exhibit significantly enhanced biological activity, including increased cellular uptake, intracellular accumulation, and interaction with critical cellular structures. Pan et al. showed that ultrasmall AuNPs (1.4 nm) induced pronounced cell death in human bone marrow cells, whereas AuNPs in the 15–80 nm range exhibited markedly lower toxicity [44]. Similarly, Chithrani et al. demonstrated that the smallest gold nanoparticles were internalized most efficiently by HeLa cervical cancer cells, which indicates that nanoparticle size directly correlates with cytotoxic potency [45]. The hydrodynamic diameters (~10–11 nm) and metallic core sizes (~7 nm) determined in the present study place the AuNPs/PAMAM G2 complexes precisely within this biologically active size regime.

Reports, particularly on gold nanoparticles reduced with ascorbic acid, often describe these systems as biocompatible, especially when they are evaluated using nonmalignant cell lines. Jayeoye et al. reported that the negligible cytotoxicity of ascorbic acid stabilized AuNPs toward RAW 264.7 macrophages [10]. These findings do not contradict present results, as nanoparticle–cell interactions are strongly dependent on both cell type and nanoparticle physicochemical characteristics. Cancer cells frequently display enhanced nanoparticle uptake [46] due to altered membrane composition [47], elevated endocytotic activity [48], and higher metabolic demand [49], which may explain the increased sensitivity of MCF-7 and Saos-2 cells to the investigated AuNPs.

Importantly, the present study demonstrates that the use of a mild, biologically relevant reducing agent, such as *L*-ascorbic acid, does not abolish the intrinsic size-driven cytotoxicity of gold nanoparticles. This observation is consistent with comprehensive reviews emphasizing that nanoparticle size and surface charge dominate biological responses, irrespective of the “green” or conventional nature of the synthesis route [29]. In this context, stabilization with low-generation PAMAM dendrimers ensures colloidal stability and controlled particle size but does not prevent nanoparticle–cell membrane interactions that entirely decrease viability in the studied cancer cell lines.

A comparison with our previous studies employing sodium citrate as a reducing agent further highlights the importance of nanoparticle size. Citrate-reduced AuNPs/PAMAM G2 complexes exhibited larger hydrodynamic diameters (~20 nm) and generally lower cytotoxicity, despite comparable surface chemistry [29]. The higher toxicity observed for the ascorbic acid-reduced systems can therefore be attributed primarily to their reduced size rather than to the specific reducing agent used. This conclusion corresponds to the literature data indicating that AuNPs below 10 nm represent the most biologically active and hereby potentially hazardous size range [29,50,51].

Collectively, these findings reinforce the concept that biocompatible synthesis conditions do not necessarily yield biologically inert nanomaterials. Instead, nanoparticle size remains the dominant parameter governing cytotoxicity, a factor that must be carefully considered when designing AuNP-based systems for biomedical and theranostic applications.

### 2.5. Presence and the Localization of AuNPs/PAMAM G2 on the Saos-2 and MCF-7 Cell Membrane

**Figure 8:**
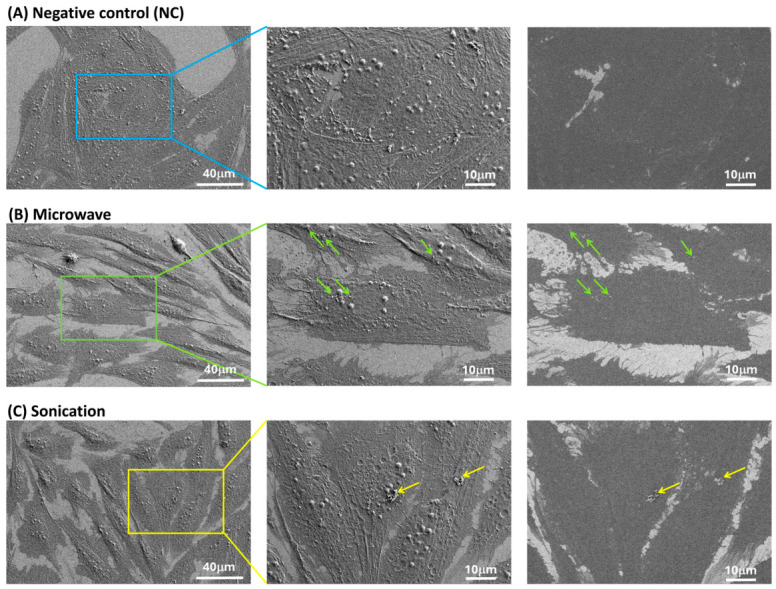
SEM images of osteosarcoma (Saos-2) control cells (**A**) and cells exposed to AuNPs/PAMAM G2 complexes synthesized by microwave irradiation (**B**) and sonication (**C**) at the EC_50_ concentration after 24 h of incubation. The left and middle panels represent images acquired using the ETD detector at magnifications of 1000× and 2500×, respectively. The right panel presents images acquired using the T1 detector at a magnification of 2500×. Green/yellow arrows indicate agglomerates of gold complexes located on or beneath the cell surface.

**Figure 9:**
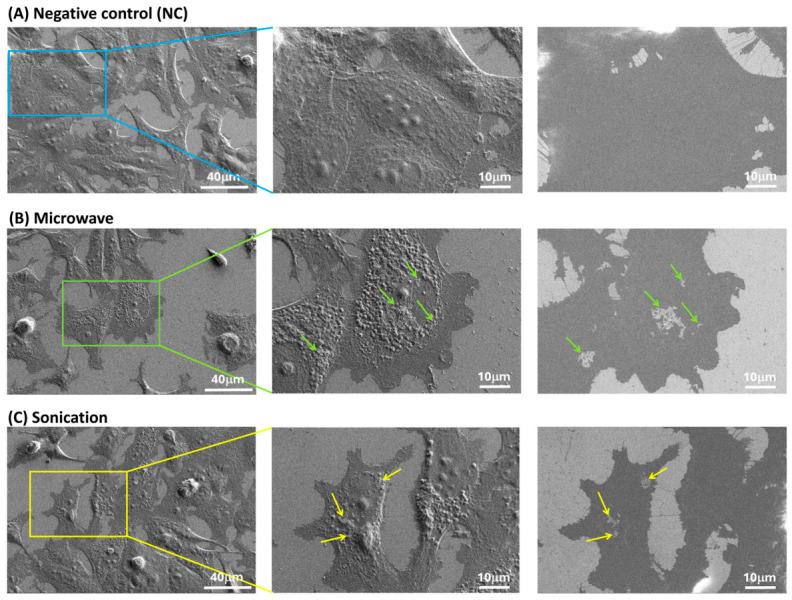
SEM images of breast cancer (MCF-7) control cells (**A**) and exposure to AuNPs/PAMAM G2 of microwave (**B**) and sonication (**C**) processes at EC_50_ concentrations for 24 h. The left and middle panels represent images acquired using the ETD detector at magnifications of 1000× and 2500×, respectively. The right panel presents images acquired using the T1 detector at a magnification of 2500×. Green/yellow arrows indicate agglomerates of gold complexes located on or beneath the cell surface.

SEM studies were performed to illustrate cell morphology and AuNPs/PAMAM G2 distribution on the cell surface. Figure 8 and Figure 9 show selected SEM images of Saos-2 and MCF-7 cell lines after treatment with gold–dendrimer complexes at EC_50_ and after 24 h of incubation. The left and middle panels present topographical images of the samples acquired using the ETD (Everhart–Thornley Detector). This detector collects secondary electrons emitted from the sample surface as a result of its interaction with the electron beam, enabling the analysis of the surface topography, morphology, and structure of the investigated objects. In contrast, the right panels of Figure 8 and Figure 9 correspond to the images shown in the middle panel but were acquired using the T1 detector, which registers backscattered electrons. The use of this detector allows for the generation of compositional contrast (Z-contrast) and the assessment of variations in the chemical composition of the sample. The areas of enhanced contrast, marked with arrows in the T1 detector images, indicate the presence and spatial distribution of metallic nanoparticles.

For the untreated cells, 70–80% confluence with numerous protrusions was observed. Cells exposed to AuNPs/PAMAM G2 exhibited decreased confluence, and most of them were detached from the surface, suggesting apoptotic or necrotic cell death. A noticeable change in cell shape to a highly elongated morphology was observed for the Saos-2 cell line (Figure 8). In all cases, predominantly agglomerated AuNPs/PAMAM G2 complexes were visible in the area of the cell nucleus.

### 2.6. Translational Barriers for the Use of AuNPs/PAMAMs in Medicine

The toxicity of the AuNPs/PAMAM system assessed in this study under cell culture conditions is a preliminary step, preceding ex vivo studies and ultimately clinical trials. Despite growing interest in the use of nanoparticles stabilized with PAMAM dendrimers, these materials have not yet reached the clinical trial phase. This is due to a number of factors, including the need to obtain representative preclinical models, carefully design clinical trials, develop specific and harmonized regulatory protocols, and incorporate non-standard financing strategies [52,53]. Additionally, the costs of synthesizing AuNPs/PAMAM on a clinical scale may pose a significant limitation to their potential diagnostic use [54]. Another key aspect of translating nanostructures into medical practice is rational design during the research and development phase. Translational barriers include the complexity of large-scale production [55,56], safety and toxicity concerns stemming from unknown long-term effects and potential accumulation, and challenges related to overcoming biological barriers such as the blood–brain barrier [57] and the body’s immune response [58]. For the intravenous administration of nanostructures, the endothelial barrier, interactions with blood cells, and changes in shear stress resulting from the presence of nanoparticles in circulation must also be considered [59]. These factors may affect the stability of nanoparticle systems in the body and also influence their potential utility.

## 3. Materials and Methods

### 3.1. Synthesis of AuNPs/PAMAM G2

Tetrachloroauric(III) acid trihydrate (HAuCl_4_·3H_2_O) and 2nd-generation ethylenediamine-core poly(amidoamine) (PAMAM) dendrimers containing 16 surface primary amine groups (20% methanolic solution; PAMAM G2) were sourced from Sigma-Aldrich (St. Louis, MO, USA). Sodium citrate was supplied by Chempur (Piekary Śląskie, Poland). Preparation of gold nanoparticles and PAMAM dendrimer conjugates has been described previously [36,37,60].

In this study, AuNPs/PAMAM G2 complexes were synthesized using sonication and microwave-assisted methods. *L*-ascorbic acid served as a reducing agent. An aqueous 1 mM PAMAM G2 solution (1 mL) was mixed with 1 mL of HAuCl_4_ solution at concentrations of 1, 2, 3, 4, or 5 mM and sonicated for 3 min at room temperature. Subsequently, 1 mL of *L*-ascorbic acid solution was added at a molar ratio corresponding to one-third of the gold concentration to reduce the Au(III) complex.

Two parallel series of colloids were prepared. One series was sonicated at 80 °C with a power output of 180 W for 60 min, whereas the other one was subjected to microwave irradiation at 800 W for 45 s. It should be noted that at lower sonication temperatures, stable AuNPs/PAMAM G2 complexes were not obtained. Subsequently, for lower microwave powers, rapidly aggregating and agglomerating colloids were obtained. A visible color change in all colloidal solutions to red was observed, confirming the formation of gold nanoparticles. According to a previous paper [36], the AuNPs/PAMAM G2 colloids were centrifuged at 12,000× *g* for 30 min. This procedure enabled the removal of extraneous ions and redox byproducts [23,61]. The resulting pellet was washed twice and resuspended in high-grade quality water.

### 3.2. Characterization of Synthesized Nanoparticles

The synthesized nanoparticles were characterized by UV–Vis spectroscopy using an Ultrospec 2100 Pro spectrophotometer (GE, Farnborough, UK). The hydrodynamic radius and size distribution were measured by dynamic light scattering (DLS) with a DynaPro NanoStar instrument (Wyatt Technology, Santa Barbara, CA, USA). Measurements were performed at 25 °C using a laser wavelength of 663 nm, laser power of 100 mW, and scattering angle of 90°. Zeta potential was determined using a Zetasizer Nano instrument (Malvern Instruments, Malvern, UK), operating at a laser wavelength of 663 nm, laser power of 4 mW, scattering angle of 173°, and temperature of 25 °C. Colloidal stability was evaluated over a period of 3 months.

Morphological characterization of AuNPs/PAMAM G2 complexes was carried out by atomic force microscopy (AFM) and scanning transmission electron microscopy (STEM). AFM imaging was performed using a MultiMode 8-HR microscope (Bruker, Billerica, MA, USA), whereas STEM observations were conducted at an accelerating voltage of 30 keV with an Apreo 2S microscope (Thermo Fisher Scientific, Waltham, MA, USA). For the AFM visualization, AuNPs/PAMAM G2 colloids at concentrations of 0.5 mg/mL with a volume of 10 µL were placed onto silicon substrates (droplet deposition procedure) and dried at room temperature. According to the previous manuscript [36], the images were acquired in tapping mode in air, using an RTESPA-300 cantilever (Bruker, USA). The AFM measurement parameters were as follows: scan size: 2 × 2 μm; resolution: 512 × 512 pixels. For STEM imaging, the nanoparticle colloids (0.5 mg/mL concentration) were placed on a Cu 300 mesh grid and dried at room temperature. Each experiment was repeated 3 times.

### 3.3. Cell Culture and Cytotoxicity Measurements (XTT Assay)

The human breast adenocarcinoma cell line MCF-7 (cat. no. HTB-22™) and the human osteosarcoma cell line Saos-2 (cat. no. HTB-85™) were purchased from ATCC (Manassas, VA, USA). Cells were maintained under sterile conditions and passaged in accordance with the supplier’s recommendations. The following reagents were obtained from ATCC (Manassas, VA, USA): McCoy’s 5A Medium, Dulbecco’s Modified Eagle Medium (DMEM), fetal bovine serum (FBS), penicillin–streptomycin solution, and 0.25% (*w*/*v*) trypsin–0.53 mM EDTA. Phosphate-buffered saline (PBS) was acquired from Gibco (Waltham, MA, USA). MCF-7 cells were routinely cultured in complete DMEM supplemented with 10% FBS, whereas Saos-2 cells were maintained in McCoy’s 5A medium containing L-glutamine and 10% FBS. Both cell lines were grown in 75 cm^2^ culture flasks at 37 °C in a humidified atmosphere with 5% CO_2_. Subculturing was carried out every 3–4 days, when cell confluence reached approximately 90%, and the first non-adherent cells appeared in the culture medium.

The cytotoxicity of the Au/PAMAM complexes was investigated using a tetrazolium salt reduction assay. The tetrazolium salt XTT (2,3-bis[2-methoxy-4-nitro-5-sulfophenyl]-2H-tetrazolium-5-carboxanilide) was purchased from Biological Industries (Cromwell, CT, USA). Cells were seeded in a 96-well plate at a density of 20,000 and 10,000 cells per well for MCF-7 and Saos-2 cell lines, respectively. After 24 h of culture, the medium was replaced with AuNPs/PAMAM suspensions prepared in serum-free medium at final concentrations of 1, 2.5, 5, 10, 15, 20, 25, and 40 μg/mL for the MCF-7 cell line and 1, 2.5, 3.5, 5, 7.5, 10, and 15 μg/mL for the Saos-2 cell line. The medium in the negative control (NC) samples was replaced with fresh serum-free medium without nanoparticles. After 24 h incubation with nanoparticles, cell viability was assessed using the XTT assay according to the manufacturer’s protocol. The absorbance was measured at 450 nm for each well using a Victor X4 microplate reader (PerkinElmer, Waltham, MA, USA). Cell viability was expressed as a percentage relative to the NC. Each experiment was repeated 3 to 4 times.

All statistical analyses were performed using OriginPro 8.0 2017 software. Group comparisons were carried out using one-way ANOVA. Levels of significance were set at * *p* < 0.01, ** *p* < 0.001, and *** *p* < 0.0001. In addition, EC_10_, EC_25_, and EC_50_ values for AuNPs/PAMAM G2 were determined, representing concentrations that reduced cell viability to 90%, 75%, and 50%, respectively. Results are presented as mean ± standard error of the mean.

### 3.4. Saos-2 and MCF-7 Cell Preparation for SEM Visualization

For SEM imaging, cells were seeded onto the glass coverslips and exposed to AuNPs/PAMAM G2 at selected concentrations for 24 h. Fixation of cells was carried out using a mixed solution of 2.5% of glutaraldehyde and 3.7% of formaldehyde for 24 h. Subsequently, the cells were dehydrated through a graded series of ethyl alcohol (50–99.8%). The preparation of cell samples exposed to AuNPs/PAMAM G2 has been described previously [20,62].

Images of fixed cells were acquired using an Apreo 2S scanning electron microscope (SEM) (Thermo Fisher Scientific, MA, USA) equipped with a secondary electron detector (Everhart–Thornley Detector—ETD) and a backscattered electron detector (T1 segmented lower in-lens detector). Imaging was performed under high-vacuum conditions at an accelerating voltage of 1 keV. To visualize the entire cells with AuNPs/PAMAM G2 or their agglomerates, magnifications of 1000× and 2500× were applied.

## 4. Conclusions

The present study demonstrates that *L*-ascorbic acid can be successfully employed as a mild and biocompatible reducing agent for the synthesis of PAMAM G2-stabilized gold nanoparticles using both sonication and microwave-assisted approaches. Under optimized conditions, this strategy enables reproducible formation of monodisperse AuNPs/PAMAM G2 colloids with hydrodynamic diameters of approximately 10–11 nm and metallic core sizes of ~7 nm, which remain colloidally stable for at least three months.

Despite the environmentally friendly and biologically relevant nature of the synthesis conditions, the obtained nanoparticles exhibit a clear, size-dependent cytotoxic effect toward MCF-7 breast adenocarcinoma and Saos-2 osteosarcoma cell lines. The present study is a continuation of our previous work [25,36,37], in which the experiments were conducted in serum-free culture medium. It is worth noting that serum proteins can form a corona around the complexes and thus mask the cytotoxic effect of the AuNPs/PAMAM G2. Therefore, further studies using complete cell culture medium are required to achieve a deeper understanding of the interactions between cells and gold-based complexes. The determined EC_10_, EC_25_, and EC_50_ values are consistent with numerous literature reports indicating that gold nanoparticles with diameters below 10 nm represent the size regime associated with enhanced cellular uptake and increased toxicological response. These findings confirm that the use of ascorbic acid as a reducing agent does not mitigate the intrinsic size-driven biological activity of ultrasmall AuNPs. 

Importantly, the cytotoxicity levels observed for AuNPs/PAMAM G2 reduced with ascorbic acid are comparable to, or slightly higher than, those previously reported for larger PAMAM-stabilized nanoparticles synthesized with sodium citrate [36]. This effect can be attributed primarily to the reduced particle size rather than to differences in surface chemistry or synthesis methodology. Results further indicate that stabilization with low- generation PAMAM dendrimers provides effective colloidal control but does not eliminate nanoparticle–cell interactions responsible for cytotoxic effects. 

Overall, this work highlights that “green” or biocompatible synthesis routes do not necessarily lead to biologically inert nanomaterials. Instead, nanoparticle size remains the dominant parameter governing cytotoxicity, regardless of the applied reducing agent. These findings are particularly relevant for the rational design of AuNP-based systems intended for biomedical and theranostic applications, where balancing size-dependent functionality and biological safety is essential. Future studies should focus on systematic surface modification and dose-controlled biological evaluation to further tailor the therapeutic window of ascorbic acid-reduced AuNPs/PAMAM complexes. 

## Figures and Tables

**Table 1:** Hydrodynamic diameter values for the AuNPs/PAMAM colloids synthesized by the sonication technique over particular periods of time, along with the percentage of mass.

Sample—Sonication	Population	Hydrodynamic Diameter Over Particular Periods of Time [nm] (%Mass)				
		24 h	1 Week	1 Month	2 Months	3 Months
1:1	Peak 1 Peak 2	1.2 (97.7%) 17.8 (2.3%)	1.8 (96.0%) 18.4 (4.0%)	- 18.0 (100%)	- 15.4 (100%)	15 (99.8%) 58.4 (0.2%)
1:2	Peak 1 Peak 2	1.0 (96.4%) 11.4 (3.6%)	- 11.4 (100%)	- 11.8 (100%)	- 12.0 (100%)	- 12.2 (100%)
1:3	Peak 1 Peak 2	10.6 (100%) -	11.0 (100%) -	11.0 (100%) -	11.6 (99.9%) 872 (0.1%)	11.4 (93.4%) 17,274 (6.6%)
1:4	Peak 1 Peak 2 Peak 3	22.4 (99.5%) 107.4 (0.5%) -	16.8 (96.9%) 93.4 (3.1%) -	18.2 (97.9%) 56.6 (2.1%) -	19.0 (99.0%) 48.8 (1.0%) -	5.2 (65.9%) 20.8 (32.6%) 9546 (1.5%)
1:5	Peak 1 Peak 2 Peak 3 Peak 4	- 7.2 (97.3%) 144.8 (2.7%) -	2.6 (94.2%) 19.0 (4.2%) 152.6 (1.6%) -	4.2 (88.1%) 17.2 (10.5%) 152.0 (1.5%) -	6.0 (91.7%) 18.2 (7.1%) 108.6 (0.8%) 1092 (0.4%)	5.6 (95.8%) 29.4 (3.7%) 136.2 (0.5%) -

**Table 2:** Hydrodynamic diameter values for the AuNPs/PAMAM colloids synthesized by microwave technique over particular periods of time, along with the percentage of mass.

Sample—Microwaves	Population	Hydrodynamic Diameter Over Particular Periods of Time [nm] (%Mass)				
		24 h	1 Week	1 Month	2 Months	3 Months
1:1	Peak 1 Peak 2	- 12.2 (100%)	1.2 (95.9%) 15.2 (4.1%)	- 13.8 (100%)	- 14.6 (100%)	- 14.8 (100%)
1:2	Peak 1	11.0 (100%)	10.4 (100%)	10.6 (100%)	10.6 (100%)	11.2 (100%)
1:3	Peak 1 Peak 2	10.0 (100%) -	10.4 (100%) -	10.4 (100%) -	10.6 (100%) -	11.0 (95.2%) 10,838 (4.8%)
1:4	Peak 1 Peak 2	4.0 (99.2%) 56.0 (0.8%)	3.8 (99.2%) 51.8 (0.8%)	3.6 (99.4%) 56.3 (0.6%)	4.4 (99.5%) 48.8 (0.5%)	3.8 (99.7%) 50.8 (0.3%)
1:5	Peak 1 Peak 2 Peak 3	- 6.4 (99.4%) 65.8 (0.6%)	- 5.6 (99.1%) 67.8 (0.9%)	1.6 (90.2%) 7.8 (9.6%) 76.4 (0.2%)	2.2 (87.0%) 8.6 (12.9%) 72.4 (0.1%)	- 7.6 (99.4%) 68.8 (0.6%)

**Table 3:** Percentage decrease in zeta potential value after 3 months.

Molar Ratio	Sonication	Microwaves
1:1	35%	37%
1:2	27%	39%
1:3	39%	15%
1:4	26%	13%
1:5	10%	17%

**Table 4:** Summary of cell viability in MCF-7 and SAOS-2 cells. EC_10_, EC_25_, and EC_50_ correspond to 90%, 75%, and 50% of control viability, respectively. For comparison, the table includes cytotoxicity levels for two cell lines treated with synthesized nanoparticles obtained using *L*-ascorbic acid as the reducing agent and sodium citrate as the previously used reducing agent, for which the results were published previously [36]. Values are expressed as mean ± standard error of the mean.

**Level of Effective Concentration**	MCF-7 Cells			
	**SONICATION** **(*L*-Ascorbic Acid)** **[µg/mL]**	**SONICATION** **(Sodium Citrate)** **[µg/mL]**	**MICROWAVE** **(*L*-Ascorbic Acid)** **[µg/mL]**	**MICROWAVE** **(Sodium Citrate)** **[µg/mL]**
EC_10_	4.2 ± 1.1	3.2 ± 0.7	6.5 ± 0.8	3.3 ± 0.7
EC_25_	10.8 ± 0.7	13.1 ± 0.3	10.0 ± 0.7	9.3 ± 0.3
EC_50_	21.9 ± 0.8	25.4 ± 0.4	15.8 ± 0.6	20 ± 0.7
	SAOS-2 cells			
	**SONICATION** **(*L*-ascorbic acid)** **[µg/mL]**	**SONICATION** **(sodium citrate)** **[µg/mL]**	**MICROWAVE** **(*L*-ascorbic acid)** **[µg/mL]**	**MICROWAVE** **(sodium citrate)** **[µg/mL]**
EC_10_	1.6 ± 0.3	2.3 ± 0.2	1.4 ± 0.2	1.4 ± 0.1
EC_25_	3.1 ± 0.3	3.9 ± 0.2	2.7 ± 0.2	2.9 ± 0.1
EC_50_	5.5 ± 0.2	6.6 ± 0.1	4.7 ± 0.3	5.4 ± 0.1

## Data Availability

Original contributions presented in this study are included in the article. Further inquiries can be directed to the corresponding author.

## Abbreviations

The following abbreviations are used in this manuscript:

AuNPs	Gold nanoparticles
DLS	Dynamic Light Scattering
DMEM	Dulbecco’s Modified Eagle Medium
EC	effective concentration
FBS	Fetal Bovine Serum
HAuCl_4_	chloroauric acid (hydrogen tetrachloroaurate)
MCF-7	human breast adenocarcinoma cell line
NC	negative control
PAMAM	polyamidoamine
PBS	Phosphate-buffered saline
Saos-2	human osteosarcoma cell line
UV–Vis	ultraviolet–visible spectroscopy
XTT	2,3-bis[2-methoxy-4-nitro-5-sulfophenyl]-2H-tetrazolium-5-carboxanilide
